# Moving from “damage-centered” research to “family-centered” participatory action research for systems change: a call to action for developmental and family scientists

**DOI:** 10.3389/fpsyg.2026.1722617

**Published:** 2026-05-28

**Authors:** Colleen K. Vesely, Bethany L. Letiecq, Xiaolu Zhang, Family Council for Equity in Education

**Affiliations:** College of Education and Human Development, George Mason University, Fairfax, VA, United States

**Keywords:** early care and education (ECE), family councils, family-centered, family-centered participatory action research, systems change

## Abstract

Nearly 20 years ago, Eve Tuck urged researchers to reconsider “damage-centered” approaches—those that document pain and “brokenness” in hopes of compelling those in power to act to remedy the suffering. Despite volumes of evidence, these narratives of brokenness have rarely yielded transformative policies; instead, they often reinforce deficit views of marginalized communities. In response, we have reexamined our own theories of change, drawing on critical frameworks and over a decade of family-centered participatory action research (FCPAR) with mothers of young children. We propose a new theory of change for scholars pursuing equity-focused systems reform in early care and education (ECE), a system that represents families’ first encounter with formal education and profoundly shapes developmental, educational, and economic outcomes. Our new theory of change is rooted in an understanding that change will take place when people can build enough power to demand it. Research has a role to play in building that power in deep partnership with those seeking their own liberation.

## Introduction

In 2009, Eve Tuck wrote a letter imploring researchers to consider the long-term impacts of “damage-centered” research—that is, research that documents peoples’ pain, suffering, and “brokenness” under the assumption that such evidence will influence those in power and move them to pass policies and laws that redress structural inequalities (p. 409). Tuck (2009a) further posited: “This kind of research operates with a flawed theory of change: it is often used to leverage reparations or resources for marginalized communities yet simultaneously reinforces and reinscribes a one-dimensional notion of these people as depleted, ruined, and hopeless” (p. 409). Tuck then urged researchers to reformulate extant theories and methods and reimagine research and its purpose, especially when conducted with communities experiencing marginalization, oppression, and social exclusion.

Tuck’s letter hit hard. For decades, we have participated in the documentation of people’s pain and suffering, centering the lived experiences of immigrant families with young children in search of a better life in America, of working parents in need of high quality, affordable childcare. But story after story (and quantitative evidence too) of familial hardship, traumatic exposures, and poor child and family outcomes when low-quality care is the only care available, coupled with extensive policy recommendations from scholars, has not motivated the passage of comprehensive policies to the benefit of those most in need (Chaudry, 2019; Morgan, 2019; Yoshikawa et al., 2013, 2017). Indeed, as Tuck (2009a) suggested, damaged-centered research does not appear to yield meaningful systems reform, but it does certainly reify people’s vulnerabilities. Upon critical reflection and dialogic engagement, we (the authors) set out to reconsider and reimagine our own theories of change and how and why we were engaging with communities experiencing marginalization. We began interrogating and critiquing the theories undergirding our research, our methods and analytical strategies, and the ways in which we were disseminating our research. And as we discuss in this article, we began implementing a family-centered participatory action research (FCPAR) approach (Letiecq et al., 2022a), and, in partnership with families rearing young children, developed a theory of change that focused on shifting unequal relationships to power and systems change. In this paper, we consider: How can developmental and family scholarship be used to not only create knowledge and inform systems, but also as a means to create *systems change* towards meeting the needs of every single child and their family? We further discuss our theory of change and conclude with a call for developmental and other child and family scholars to likewise rethink their theories of change as we collectively work to ensure every family thrives in the United States and around the globe.

### Structural inequities in early care and education and the limits of damage-centered research

A review of the early care and education (ECE) literature makes clear that families experiencing structural oppression and economic marginalization disproportionately encounter obstacles accessing high-quality ECE in the U.S. These obstacles are many, including limited information, unmet language and transportation needs, paperwork burdens, and discriminatory bureaucratic encounters (Copeland et al., 2024, 2025; Friedman-Krauss and Barnett, 2020; Park and McHugh, 2014). But the system itself is an obstacle to ECE. Indeed, the U.S. ECE system has been described as a complex system of systems that is challenging to navigate and to align services to needs, especially among families who work nonstandard hours or earn too much to qualify for subsidies but not enough to afford unsubsidized high-quality care (Ferreira van Leer et al., 2021; Ullrich et al., 2019). Even among families who qualify for subsidized ECE, inadequate slots to meet the demand and waitlists are the norm (Bouek, 2023; Copeland et al., 2025). In the absence of guaranteed access to universal ECE, inequities in developmental, educational, and familial opportunities and outcomes—at the intersections of race, class, gender, relationship status, and immigration status—emerge in early childhood and persist across the life course (Garcia and Weiss, 2017; Iruka et al., 2022).

Mitigating and redressing these structural inequities should not require the production of more “damage-centered” research (Tuck, 2009a). Nor should interventionists continue to demand that families master how to navigate complex ECE systems to improve their children’s opportunity structure and lot in life. When systems, including their laws, policies, rules and regulations, are undergirded by research that emphasize failure, risk, or deficiency, or place the blame of ECE access challenges at the feet of families, such studies embed those narratives into the very structures intended to support children and families (McWayne et al., 2021; Russell et al., 2022; Souto-Manning, 2023).

This paper serves as an invitation to developmental and family theorists, researchers, and practitioners, those positioned to influence and shape knowledge, practice, and policy—to reconsider their theories of change and their relationships with the families they wish to serve. Rather than theorizing “about” or conducting research “on” families, for example, we invite scholars to theorize, research, and practice their craft alongside and “with” families experiencing structural marginalization, oppression, and social exclusion. We argue that by partnering strategically with parents, caregivers, and families as experts and co-producers of knowledge about necessary ECE system reforms, we both democratize knowledge and strengthen our collective capacity for organization, mobilization, and action-taking to reform ECE systems and improve ECE access for all (Kapoor, 2019).

Informed by critical theories (Collins, 2019; Freire, 2000; Tuck, 2009a; Wilson, 2008) and drawing on over a decade of our team’s community-based and family-centered participatory action research (FCPAR) with mothers of young children (Letiecq et al., 2022a; Wallerstein et al., 2018), we detail a new theory of change or conceptual framework for scholars committed to using research for systems change, equity and justice. We focus on the ECE system, especially in terms of access, as this system represents children’s and families’ first connection to formal education, and is critical to the promotion of positive developmental, educational, health, economic, and familial outcomes (Bai et al., 2020) especially among marginalized and minoritized children (Bustamante et al., 2021). Specifically, we provide a brief history of critical empirical work in developmental and family science–work that challenges the historically Eurocentric perspective of child development and family relations. We then clarify why a particular focus on ECE systems change work is necessary, and how families are central to this change. And, finally, we detail aspects of a family-led theory of change for building equitable systems.

Importantly, this work was born out of our FCPAR efforts. FCPAR falls under the umbrella of community-based participatory action research (CBPAR; Wallerstein et al., 2018) and is an approach to research that is based upon a trusted partnership between researchers and families, where families experiencing marginalization drive all aspects of the research enterprise (Letiecq et al., 2022a). In this instance, our theory of change work has been co-led by our strategic community partners, the Family Council for Equity in Education (“Family Council”). The Family Council is a community advisory board (CAB) composed of eight parents and caregivers from economically disinvested communities in the northern Virginia region. They represent diverse intersectional identities across race (Black, White, multiracial), ethnicity (Latine), gender (male/female/nonbinary), relational status (e.g., married, partnered, single parent), nationality (US/Central America/Peru), ability (parents with disabilities and parents of children with disabilities), and language (Spanish/English). We have been working in partnership for approximately 3 years.

### Critical developmental theories on structural inequities and children’s development

Our work with the Family Council is undergirded by intersectionality, a critical social theory and praxis that locates individuals, families, and scholars within a matrix of domination and oppression, where interlinked inequities resulting from colonialism, capitalism, racism, sexism, and nationalism are maintained at a structural level (Author et al., 2023; Collins, 2019; Crenshaw, 1989; Few-Demo, 2014). Intersectionality interrogates how systems, including ECE systems, operate to reinforce unequal power relations and structural inequities to the harms of those experiencing social, legal, and economic marginalization (Bailey et al., 2021; Young, 1990). In addition, there is an important history of developmental science scholarship that informs the potential transformation of early childhood systems.

We are particularly influenced by critical child development scholars who in the latter part of the 20th century began to challenge the Eurocentric focus and framing of developmental science. Eurocentrism, a concept introduced by Samir Amin, refers to the ideology of European cultural superiority and the tendency to interpret the world through a European capitalist lens, often at the expense of non-European societies (Olende, 2019). From the early modernization paradigm to the more recent neoliberal framework, mainstream developmental theories have remained rooted almost exclusively in the historical and social experiences of a small number of Western industrialized nations (Brohman, 1995).

These Eurocentric developmental theories inform policy and practice, as well as the general cultural ethos regarding poor families and families of color, which critical scholars continue to unravel today (Bailey et al., 2021; Copland et al., 2024; Iruka et al., 2022). At best these developmental theories ignored families who fall outside of the White European Standard North American Family (SNAF) (Smith, 1993), and at worst, painted a picture of non-SNAF and families of color as “inherently wrong or deficient,” labeling them at risk (Johnson et al., 2025, p. 5). For example, Piaget’s theory of cognitive development, often celebrated as a universal account of how children learn, reflects this Eurocentric worldview as he presented his stages as “natural laws” of development. Piaget positioned the Western child, educated within European schooling traditions and cultural assumptions, as the universal benchmark for all children. This universalism, which was readily embraced in postwar contexts that emphasized human rights and meritocracy, effectively erased cultural variations in how childhood and learning were understood globally (Blundell, 2017). Sardar (1998) emphasized that development itself is a Eurocentric concept. He argued that the true power of the West lies not merely in its economic growth or technological advances, but in its ability to define the very terms and frameworks of development. Tucker (1999) argued that “development” is a Eurocentric discourse and a myth that legitimizes Western dominance. Its real power lies in defining reality, shaping how we think about progress, silencing other cultural models, and ignoring structurally oppressive forces that differentially impact children and families in an unequal and racialized society.

As Johnson et al. (2025) highlighted, “Developmental science has historically been leveraged to justify racial subordination of youth and reinforce White supremacy in developmental science…at three levels of theory: description, explanation, and prediction” (p. 5). Through deficit and “damage-centered” descriptions, explanations, and predictions, developmental science perpetuated colonial and oppressive dynamics by producing knowledge that justified intervention on marginalized communities rather than partnership with them to redress the structural features of oppression and social exclusion (Tuck, 2009a).

Standing in critique of these Eurocentric theories were scholars of color who posited the ways in which structures, rather than “culture” or individual choices, regulated and controlled Black and immigrant families’ lives and in turn, children’s development. These scholars (e.g., Burton and Jarrett, 2004; Garcia Coll et al., 1996; Spencer, 1997), whose work continues to inform the child development field today, put forth theoretical models that centered the developmental experiences of children of color in context, highlighting families’ strengths, and families’ adaptation in the face of deficit-oriented, structurally-oppressive systems informed by Eurocentric child and family research.

Garcia Coll et al. (1996), in response to ecological developmental theories that were devoid of systemic inequities, articulated a new Integrative Model of Child Development that intentionally focused on children of color at the intersection of race, ethnicity, culture and social class. The integrative model highlighted social position, discrimination, and racialized segregation connected to systems, coupled with family resistance vis-a-vis the development of adaptive cultures in shaping the developmental pathways of racially and ethnically minoritized children. Importantly, this framework continues to be used extensively by developmental scientists today given its on-going applicability to understanding children’s development in a racialized and unequal society (Perez-Brena et al., 2018). This framework was a call for moving beyond an individualistic, Western-centric lens to address complex, socially-constructed challenges. It was also a call to reject deficit-based viewpoints and move towards empowering communities historically seen as “other” to identify and articulate their own strengths and define developmental pathways that honor their lived realities.

Critical developmental theories, like Garcia Coll et al.’ (1996) integrative model, have shaped developmental scholarship over the last several decades to be more inclusive, strengths-based, and critical, highlighting the structural conditions and constraints and cultural adaptive facilitators connected to children’s development and families’ experiences (Johnson et al., 2025). Still, even with a depth of understanding of these pathways and the structural forces that regulate the lives of children and youth who experience structural marginalization, programs, institutions, and systems shaped by policies and laws continue to leave children and families’ needs unmet. Critical theories help us understand families’ strengths in the face of oppression, and critical methods guide us to truly center these strengths as we co-construct knowledge with communities. As Tuck (2009a) described, moving beyond damage-centered research towards what she calls desire-based research, foregrounds communities’ strengths, resilience, knowledge, and aspirations and emphasizes people’s full humanity. Co-constructing knowledge with families most impacted by the system–families in Garcia Coll et al.’ (1996) model who were forced to adapt to an oppressive system– is imperative for *transforming* systems to equitably support children’s development (Author, 2025).

### Critical family theorizing on structural inequities and family relations

Alongside the emergence of critical developmental theories, which interrogate how racism, classism, sexism, and other systems of oppression shape developmental processes and outcomes across the life course, have been critical family theories, which examine how those same systems organize, regulate, and stratify family life. Together, critical developmental and family scholarship underscore that children do not develop apart from families, nor do families operate outside of the sociopolitical context (Coll et al., 1996; Collins, 1994; McLoyd, 1998). Rather, developmental trajectories are co-constructed within family systems that are themselves structured by race, class, gender, legal status, and family laws and policies (Vesely et al., 2019a). These structuring elements condition and constrain family life, including children’s access to ECE, and, subsequently, their developmental outcomes (Vesely et al., 2021; Letiecq et al., 2022b; Jensen and Sanner, 2021; Sanner et al., 2024).

Critical family scholars argue that the traditional two-parent married nuclear family system with a breadwinning father and stay-at-home mother, referred to as the Standard North American Family (SNAF; Smith, 1993), has been held up by U. S. society and by many discourses as the normative and optimal context for child development (Seita, 2014; Letiecq, 2019, 2024). This family form has been held up as best for rearing children and maintaining social order, to the disadvantaging of all other family forms (Letiecq, 2019, 2024). Developmental science has not been immune to this normativity (Heyes, 2024). Much of the field’s foundational research, often conducted with White middle class, married heterosexual families, has implicitly treated the SNAF as the baseline against which other families are compared (Allen, 2000). In turn, deviations from this model (e.g., single-parent, multigenerational, cohabiting, or immigrant families) have too often been framed in terms of risk, instability, or deficit rather than structural constraint or adaptive strength (McLanahan et al., 2013; Osborne and McLanahan, 2007).

This valorization of the SNAF has been fortified by over 1,100 benefits, rights, and protections specifically designed for SNAFs that are often not accessible to other families (e.g., single parents, cohabiting couples; Letiecq, 2019). Such benefits include access to spousal health care benefits, social security benefits, and, as Brown (2021) details, tax benefits that support the generation of familial wealth. Those substantial benefits, rights, and protections, sanctioned by the state, serve to bolster and privilege SNAFs while other policies, laws, rules, and regulations (e.g., Temporary Aid for Needy Families; social programs with work requirements and drug testing) serve to denigrate, delegitimize, and dehumanize non-SNAFs, including families headed by single mothers, families of color, and immigrant families (Letiecq et al., 2022a; Floyd et al., 2021; Parolin, 2021). Thus, structural inequities embedded in family laws and policies become developmental inequities, shaping children’s opportunity structures and well-being from the earliest years.

Family scholars further contend that the systematic SNAF privileging over all other family forms explains, in part, why the U.S. lags so far behind other wealthy, industrialized nations in the provision of universal ECE and paid family leave benefits (Gerson, 2010; Smith, 1993). Under the dictates of White heteropatriarchal supremacy, the U.S. has positioned the stay-at-home mother as ideal and essential for child well-being, while mothers in the labor force have been cast as selfish or insufficiently devoted to their children (Calarco, 2024). Developmental research emphasizing maternal sensitivity, attachment, and early caregiving (Minnotte, 2023), often without attending to structural labor market constraints, has at times been used to reinforce these cultural narratives. Conversely, low-income mothers, and especially those who are single parents, have been vilified in the U.S. as deficient, immoral, and unfit. Under welfare reform and other social policies, poor single mothers are forced to work for their benefits. Ironically, they are not guaranteed high-quality ECE. The contradiction is stark: mothers experiencing economic marginalization are required to work, yet the developmental infrastructure necessary to support children’s well-being remains fragmented and inaccessible.

This privileging of one family form by law, policy, and practice conditions and constrains family functioning in racialized and gendered ways. For example, it places caregiving responsibilities squarely on the shoulders of mothers and then differentially conditions mothers’ labor market participation as a function of race, class, and gender (Folbre, 2021). These structural arrangements influence proximal processes central to developmental science, parent–child interactions, stress exposure, time use, and access to stimulating learning environments (Lareau, 2011; Shonkoff and Phillips, 2000). Family scholars posit that the privileging of traditional nuclear families in the U.S.–where (White and wealthy) stay-at-home mothers provide the lion’s share of ECE for their children–is a primary reason for the modern-era piecemeal ECE system of systems that exists today to service the care needs of those “other” mothers (Calarco, 2024).

Understanding the complex ways that families are privileged and disadvantaged at a structural level and why (i.e., to uphold the SNAF as best family model) is essential to understanding why damage-centered research has not been able to move the needle to advance significant improvements to the ECE systems in decades. Indeed, damage-centered research helps to perpetuate “deficit narratives” among non-SNAF families, justifying among some the rationale for promoting traditional two-parent married families as best for children and society (Letiecq, 2019, 2024).

### Early care and education: key developmental context ripe for systems change

High-quality early care and education (ECE) can disrupt structural inequities and unequal outcomes from early childhood through adulthood (Bai et al., 2020; Bustamante et al., 2021; Schoch et al., 2023). Beyond educational and health benefits, ECE provides responsive educator relationships, developmentally appropriate curricula, and a bridge for families into formal education (Schoch et al., 2023). Yet, persistent beliefs that mothers belong at home and limited political will to fund universal ECE have produced a fragmented “system of systems” or “non-system” (Doromal et al., 2023; Kagan and Cohen, 1997) that fails to meet demand and perpetuates barriers tied to wealth, language, transportation, paperwork, and discrimination (Copeland et al., 2024, 2025; Ferreira van Leer et al., 2021; Friedman-Krauss and Barnett, 2020).

Despite consensus on ECE’s importance—especially for children experiencing structural marginalization—U. S. federal, state, and local governments have been unable or unwilling to reduce systemic inequities or complexity (Bassok et al., 2018). In the absence of a universal system, families must navigate a complex, publicly and privately funded “non-system,” placing disproportionate burdens on those facing structural marginalization and contributing to poorer child outcomes across race, class, gender, relationship, and immigration lines (Schoch et al., 2023; Yazejian et al., 2015).

We focus on the ECE system because of its clear connections to increasing educational equity by interrupting disparate outcomes among children and families who experience structural marginalization. Moreover, as “democracy and education [are] inseparably interconnected,” the more people have access to education and in turn, knowledge, the more they can participate in the democratic rule-making establishment (Moss, 2021). ECE is where children, parents, and families initially connect to formal education, and ECE has a long sociopolitical history as an important contributor to building democracy (see Reggio Emilia as a collective ECE democratic project; Moss, 2021).

ECE is ripe for systems change. And our work with the Family Council, led by parents with young children who experience structural marginalization, suggests that families want this change *desperately* as it is linked to their very liberation (Collins, 2019). Through our FCPAR partnership, we have spent nearly three years documenting families’ frustrations: not knowing about the various ECE programs that could benefit their children; only learning about ECE offerings after their children were too old; being priced out of the market; utilizing substandard or unsafe care. Even families eligible for publicly-financed ECE often cannot find open slots. These kinds of challenges are commonplace (Vesely, 2013; Vesely et al., 2021; Copeland et al., 2024, 2025; Guevara, 2024).

These systemic issues are exacerbated by the lack of user-friendly supports to help families navigate the ECE landscape (Ullrich et al., 2019). In their absence, families facing multiple marginalizations must make critical care decisions with incomplete information, often leading to compromised care or no care at all (Park and McHugh, 2014; Ullrich et al., 2019). National data highlight the scope of this inequity: only 35% of four-year-olds are enrolled in state-funded ECE, and just 15% of subsidy-eligible families receive assistance (National Institute for Early Education Research, 2023). Inflexible, low-wage work; housing instability; and a hostile, anti-immigrant policy climate further constrain access (Letiecq et al., 2022a; Vesely et al., 2021; Bovell-Ammon et al., 2021). The resulting burdens are profound—many parents, especially women, are forced to reduce hours, leave jobs, or relocate—undermining family stability, economic security, and social mobility (Bleiweis et al., 2020). As we consider the most recent 30 years of critical developmental science *and* the inequities that continue to persist in early childhood, inclusive of early childhood systems, as scholars, we must interrogate: how can developmental and family scholarship be used to not only create knowledge and inform systems, but also as a means to create *systems change* towards meeting the needs of every single child and their family?

### Families hold the key to ECE systems change: FCPAR provides an approach

Intersectionality interrogates how systems, including ECE systems, operate to reinforce unequal power relations and structural inequities (Bailey et al., 2021; Young, 1990). This theory and praxis posits that those experiencing structural marginalization: (1) are experts on how oppression shows up in their everyday lives, and (2) are most knowledgeable about and motivated to advance their liberation from oppression (Collins, 2019; Skelton-Wilson et al., 2021). True liberation from oppression comes from everyday people gaining agency, shifting power, and participating in the transformation of systems that continue to oppress (Freire, 2000). Central to transformative change is knowledge democratization, organization, and mobilization, or spreading knowledge across the general population and organizing for action-taking and structural change (Kapoor, 2019; Smith et al., 2018; Wilson, 2008).

Developmental and family research can and should play a key role in generating critical theories, engaging in knowledge democratization, and supporting systems change to advance people’s liberation from oppression. To do this work, community-based participatory action research (CBPAR) approaches (among others) are essential, connecting researchers and marginalized communities in partnership to conduct research that matters to people’s everyday lives and holds the possibility of transforming systems and structures (Letiecq et al., 2022a; Wallerstein et al., 2018). CBPAR approaches redefine the relationship between the researchers and “the researched,” and engage marginalized families and communities as strategic partners, co-researchers, and change agents—rather than merely research “subjects” or sources of extractable knowledge (Letiecq et al., 2022a; Guerrero Ramirez et al., 2022; Smith et al., 2018; Wallerstein et al., 2018).

In 2022, our research team introduced a derivation of CBPAR that we call “Family-Centered” PAR (FCPAR; Letiecq et al., 2022a). FCPAR, like other community-based approaches, works to shift power relations such that community members drive all aspects of the research process, from question formation and participant recruitment to analysis and dissemination. However, FCPAR differs from CBPAR in that families are centered in the research, often as the unit of analysis, rather than individuals or communities. Using an FCPAR approach, we seek to democratize knowledge about family life and how that knowledge is created and shared, recognizing that families not only experience structural marginalization in social systems but also in research (Smith et al., 2018). Thus, it is imperative that our research is conducted in partnership with families, is of, by and for the people, and that families experiencing structural marginalization are the direct beneficiaries of the research (Letiecq et al., 2022a). As FCPAR scholars, we agree “that people–especially those who have experienced historic oppression—hold deep knowledge about their lives and experiences, and should help shape the questions, [and] frame the interpretations of research,” (p. 458) (Torre and Fine, 2006). Importantly, we are committed to taking action that is directed by marginalized families to ensure our collective FCPAR efforts are meaningful, wanted, transformative, and sustained over time (Letiecq et al., 2022a; Wallerstein et al., 2018).

In 2023, using an FCPAR approach, we helped to stand up the Family Council, with a mission to transform the ECE system to promote equity and access. Our work together is located in northern Virginia, just minutes from our nation’s capital, where power and wealth surround “islands of disadvantage” with insufficient ECE access (Woolf et al., 2023). Early on, the Family Council established leadership and governance structures, as well as long-term goals for ECE systems change. As university partners, we have been invited by the Family Council to facilitate strategic planning and research agenda setting, and to provide resources and support necessary for them to carry out their work (e.g., locating meeting spaces, providing interpretation, translating documents). Together, over 3 years of working collectively, we have conducted focus groups and in-depth interviews, co-analyzed data, attended workshops and webinars, and co-created and co-presented a theory of systems change detailed below.

### Theory of change: building power, democratizing knowledge, mobilizing for action

**Figure 1:**
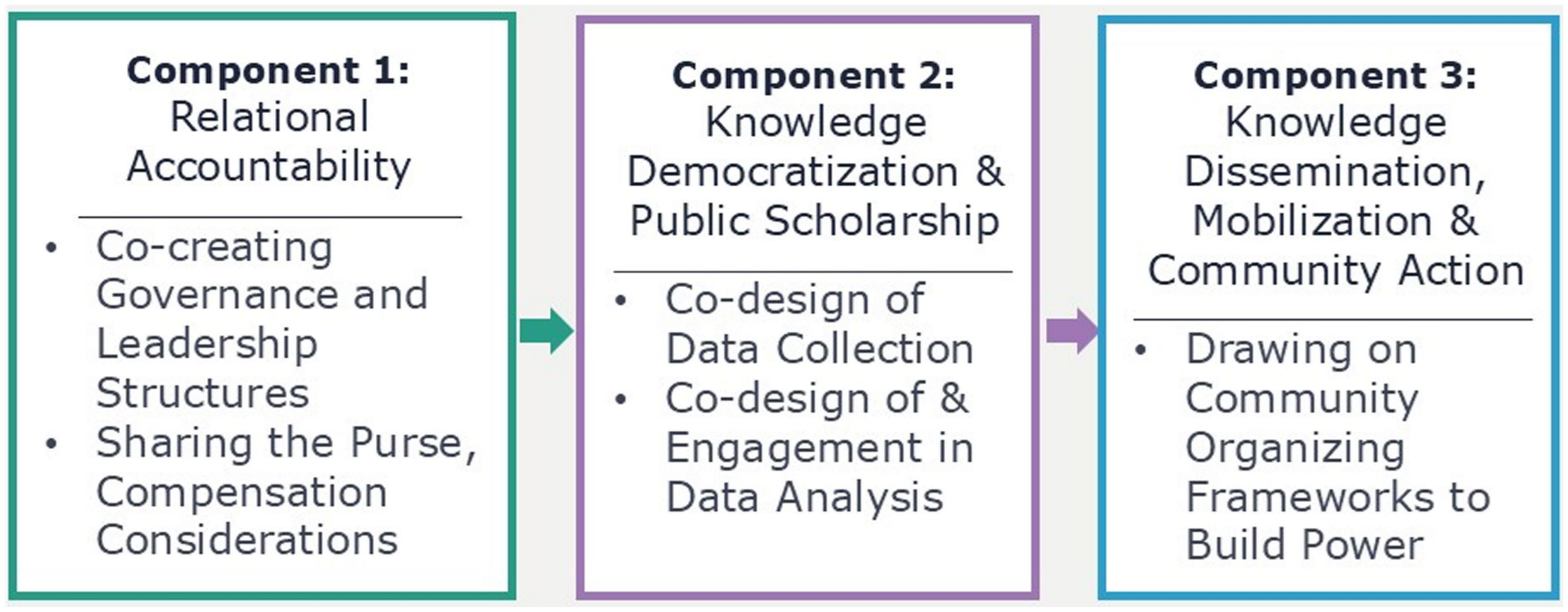
Theory of change for moving from damage-centered to family-centered research for systems change.

Our theory of change (see Figure 1) seeks to expand marginalized families’ access to high-quality early care and education (ECE)—both formal and informal—by democratizing information about the system and its services. Knowledge access generates power; through community-based efforts to connect and organize families, we aim to foster collective mobilization and systems change from the ground up. Because bureaucratic resistance often limits innovation from within, our approach builds pathways for change from outside the ECE system—requiring institutions to respond to the data produced by and actions taken by families themselves. Challenging entrenched power imbalances in ECE (Iruka et al., 2022) demands critical, reflexive practice among those closest to power, recognition of how systems reproduce privilege and oppression (Collins, 2019; Young, 1990), and a commitment to new ways of knowing and doing that center the transformative agency of marginalized families in systems not built for or with them.

Our theory of change draws on intersectionality as a critical social theory (Collins, 2019; Crenshaw, 1989), principles of FCPAR, and critical ways of knowing and methodologies (Smith et al., 2018; Wilson, 2008) and centers three inter-related and non-linear processes: (1) commit to relational accountability and building power; (2) commit to knowledge democratization and public scholarship; and (3) commit to take action through knowledge dissemination and social mobilization. This theory of change rests on the premise that meaningful transformation occurs when communities build the collective power to demand it. Research, therefore, must serve as a tool for cultivating that power in authentic partnership with those pursuing their own liberation.

### Component 1: relational accountability and building co-equal power

Calls for family- and community-driven ECE systems change mark a paradigm shift (e.g., Bailey et al., 2021). This shift acknowledges: (1) systems perpetuate unequal power and racial disparities (Young, 1990); *and*, (2) transformation must be led by those most affected by oppression (Collins, 2019). Using FCPAR, we envision a role for researchers in this transformation, working in partnership with families to co-construct and democratize knowledge and take action together for systems change. An essential ingredient of this work is the establishment of authentic community-university-system partnerships characterized by *relational accountability* (Wilson, 2008). Relational accountability recognizes that families who are experiencing marginalization are experts on how oppression manifests in their everyday lives, how systems are misaligned to their needs/goals, and how systems must change to meet their needs. Those closest to power (both researchers and agency workers) must be accountable to the families they serve, recognizing that families hold knowledge and are the most motivated for systems change in order to secure their own and their children’s liberation from oppression (Collins, 2019; Letiecq et al., 2022a, b; Young, 1990).

Relational accountability, or responsibility to the land, community and future generations, is foundational in Indigenous and decolonizing methodologies (Wilson, 2008) as well as FCPAR, and reflects “core indigenous values of integrity, respect, humility, and reciprocity” (David-Chavez et al., 2024, p.10). In his book *Research is Ceremony*, Cree scholar Shawn Wilson (2008) defines relational accountability in research as: “in essence…that the methodology needs to be based in a community context (be relational) and has to demonstrate respect, reciprocity and responsibility (be accountable as it is put into action)” (p. 99). Below, we illustrate these components of relational accountability with our FCPAR work with the Family Council. We also draw upon our work in partnership with other Councils or Community Advisory Boards (CABs) over the last decade (Hernandez Dubon et al., 2024; Letiecq et al., 2019; Letiecq et al., 2022a; Vesely et al., 2017, 2019b, 2021, 2023, 2025). These examples provide concrete strategies that other researchers or practitioners may find useful when seeking to practice relational accountability when engaging in family-centered and family-led PAR for systems change.

The work of relational accountability may seem straightforward, but working within systems can challenge individual commitments and how these commitments “show up” in this work. For example, our Family Council partners have shared stories about government agency staff who asserted they wanted to “learn from the community” and “build engaged relationships” but ultimately were unable or unwilling to authentically center family voices in their agency work. Family members on the Council described how, in their experience, systems often invoked the value of their lived experience to inform policy, yet staff could become dismissive or simply disregard family input when it conflicted with predetermined agendas. Rather than working *with* families to schedule meetings at times convenient for families or co-develop meeting agendas, Family Council members have shared experiences of subordination, where agency staff needs and goals took priority over theirs. FCPAR practitioners must be cognizant of these unequal relations and power dynamics, as well as long histories of mistreatment and extractive practices that have harmed families and promulgated mistrust, rather than co-equal power and equity (Smith, 1999).

#### Co-creating family council governance and leadership structures

To build relational accountability into FCPAR work, we recommend co-researchers consider the governance and leadership structure of the CAB. Although governance structures can vary depending on the needs and interests of community members, in our case, it was critical for the Family Council to develop a clear governance and leadership structure to redress histories of extraction and feelings of mistrust. After researching extant governance models and deliberating, the Family Council established bylaws limiting membership to families and caregivers rearing young children and specifying member roles and responsibilities. University researchers and other advisors or agency officials were included in the bylaws as ex officio, non-voting members, which clearly delineated power on the council. As FCPAR partners, the university research team supported the Family Council in holding officer elections, drafting bylaws and goals, and determining meeting rhythms —monthly, alternating between in-person and online formats. Meetings were scheduled around community availability, with our university research team providing logistical support (e.g., food, transportation, interpretation) so Family Council members could focus on their leadership roles. Co-constructed agendas, built by the Family Council president and executive team, centered on issues prioritized by the community.

As early childhood practitioners and researchers seek to center families lived experiences in their work, structures that vest power in Family Councils or CABs are essential. Such community-led boards can serve as checks on institutional power, shift decision-making back to families, and foster conditions for authentic, trusting relationships between communities, systems, and university researchers—often on terms set by the community. For practitioners and researchers, this work of shifting power structurally requires sustained engagement, active listening, open communication feedback loops, and accountability as relationships evolve, change, and grow (Gerlach, 2018). Ongoing reflexivity is central to this work, including regular check-ins with all involved. Examining and discussing Family Council dynamics—both internally and in relation to university and system partners—can meaningfully ensure alignment with community goals and guard against the reassertion of institutional power (Tynan, 2023).

#### Sharing the purse: compensation considerations

In addition to supporting the development of board leadership and building governance structures that are controlled by “the people,” another important consideration regarding relational accountability is “sharing the purse” with community partners. Grounded in FCPAR principles, we are committed to building research budgets that are meaningfully and equitably shared with our partners. With the Family Council, for example, our university research team co-developed multiple grant proposals where we committed roughly half of the budget to the community in the form of compensation for time and expertise (participant support), childcare, transportation, and programming. These resources sustain Council members’ long-term engagement, are meaningful to members, and demonstrate material commitment to shared values and shared power.

However, there are many challenges to this kind of material support, especially when working through complex and often burdensome university or governmental systems and rules that were not developed for this kind of participant support. For example, our community meetings can run 2–3 h in length. Collectively, the Council and our research team determined that participants would be compensated $100 per session, but, according to university rules, we can only use VISA gift cards for payment. Council members do not prefer this method—cards get lost, cards do not always work—and members are limited in what they can purchase with the cards. While gift cards are not cash, they are treated as cash. Thus, there are also antiquated tax issues when compensating Council or CAB members (see Waltz et al., 2023) that only add to compensation challenges. By building and maintaining structures that guard against institutional dominance, committing significant resources directly to families, and working together to redress compensation challenges, Family Councils can serve as vital checks on power and hold systems accountable while advancing family-led systems change. For those closest to power, this work requires sustained humility, reflexivity, and inter-relational accountability to ensure that systems transformation is truly community driven.

### Component 2: knowledge democratization through public scholarship

A second key component of using FCPAR to advance family-led systems change is democratizing knowledge through our engagement in public scholarship (Ward, 2012). Here we focus specifically on the co-construction and co-ownership of data with researchers and communities. This component recognizes that the researcher is not the (sole) owner or producer of knowledge, that knowledge arises through relations, or in partnerships, and that the sharing of knowledge should be co-determined. Importantly, the way you present or “tell” knowledge must honor those relations and be dictated or approved by community partners.

#### Co-design of data collection

Our university research team has collaborated with multiple communities to co-design and implement both quantitative and qualitative studies. Using an FCPAR approach, it is imperative that the collective determines the study goals, research questions, and appropriate methods to use in collecting data. With the Family Council, we began our research engagement by developing and conducting in-depth interviews and focus groups. The Council decided to pursue *interviewer-assisted surveys*, an evidence-based strategy for engaging structurally marginalized populations that the Council believes best suits their community needs (Letiecq et al., 2019; Vesely et al., 2024). Rather than beginning with predetermined methods, the research process is community-driven and unfolds in multiple, iterative phases.

Each phase of our work with the Family Council has typically begun with Council members identifying priorities rooted in families lived experiences. These priorities, which have consistently included early care and education (Vesely et al., 2021, 2024), alongside housing (Vesely et al., 2019b), food insecurity (Letiecq et al., 2022a), employment (Vesely et al., 2024), parenting (Vesely et al., 2024), and mental health (Letiecq et al., 2019), have shaped the scope of inquiry. Among our immigrant community partners, concerns about safety, detention, and deportation also have been particularly salient. While community members articulate focal issues, university researchers may suggest complementary areas of exploration to situate local concerns within broader systems.

In our most recent engagement, we grounded survey development in qualitative inquiry. We began with in-depth interviews and focus groups with Family Council members to refine constructs and clarify how priorities are experienced in daily life. Qualitative data were useful to inform the adaptation and selection of survey measures. In workshop sessions, Family Council members and university researchers reviewed existing instruments, such as items from the publicly available Rapid Assessment of Pandemic Impacts on Development (RAPID) survey, and assessed their relevance, clarity, and cultural responsiveness. Structured feedback was gathered through cognitive interviews, focus groups, and rank-ordering exercises.

As we have done with other FCPAR projects, the finalized surveys will be translated, and back-translated into multiple languages, and piloted with Family Council members. Because we are working with research participants who have had limited access to formal education, literacy levels may be low. Using an interviewer-assisted survey has been meaningful in the past as it allowed us to clarify items in real time, further enhancing accessibility and accuracy. Family Council members participated in a modified research ethics training and typically assisted with recruitment—receiving compensation for their efforts—while university researchers administered the survey to community members.

Across phases, this FCPAR approach intentionally links lived experience to qualitative and quantitative measurement, ensuring that data collection tools reflect community priorities while producing actionable evidence to inform systems-level change.

#### Co-design of and engagement in data analysis

Across FCPAR projects, we have utilized a variety of analytical strategies in partnership with Council or CAB members (Vesely et al., 2019b, 2021, 2024). For the current project, the university research team has assumed primary responsibility for data cleaning and management, while Family Council members have informed analyses and interpretation. Given institutional constraints and the realities of working with undocumented participants whose primary language is Spanish, which limited their opportunity to engage with standard university research ethics training, our research team adapted our community data analysis procedures to ensure compliance with the university Institutional Review Board (IRB) while remaining true to our FCPAR commitments. To support this, we developed a tailored research ethics training specifically for community members involved in data analysis. Additionally, community members never retained any data; all data they worked with were cleaned, anonymized, and limited to the scope that would be shared in a published manuscript. With qualitative data, the university team engaged in community coding (see Vesely et al., 2019b, 2021, 2024) with the Family Council to ensure accuracy of interpretation, working through separate passages of data together, rather than full transcripts.

On multiple occasions, our initial analyses would have yielded incorrect conclusions had the Council not been involved. We detail the specifics of these *community coding strategies* elsewhere (Vesely et al., 2019b). The Family Council advised the research team on how best to present findings to the broader community. In one project, for example, the Council guided the creation of data displays for a gallery walk, during which Family Council members led small groups of community members through the gallery walk, and concluded the meeting with a facilitated community discussion regarding collective action planning, informed by the data. Beyond community meetings—which remain the primary venues for initial dissemination—Family Council members have co-presented findings at local and national conferences and co-authored scholarly manuscripts. While data management resides with the university research team due to Institutional Review Board (IRB) requirements and community capacity constraints, data are co-owned by the Family Council and the research team, with commitments in place to secure Council permissions prior to publications and presentations. (In other cases, the community may assert full ownership of the data, a decision that should be determined prior to research engagement.)

Through co-construction and co-analysis of data, families and communities move from being research “subjects” to becoming knowledge holders and decision-makers who can leverage evidence to advance their priorities and hold systems accountable. This democratization of knowledge not only strengthens the rigor and relevance of research but also redistributes power by ensuring that data are generated, interpreted, and used in ways that honor families lived experiences and collective wisdom. FCPAR thus offers a pathway for building community capacity, disrupting extractive research practices, and positioning families as equal partners in data-driven and evidence-based systems change. Ultimately, when data are shared as a community resource rather than solely owned as an academic product, they become a catalyst for organizing, advocacy, and meaningful systems transformation.

### Component 3: knowledge dissemination, mobilization, and community action

Democratizing knowledge through public scholarship, communities gain not only data that are relevant to their goals, but also legitimacy and authority to use evidence as a tool for collective action. Unlike data generated and held exclusively by external researchers or institutions, community-owned data are grounded in families lived experiences and aligned with community-identified priorities (Emmons et al., 2024). This grounding enhances the credibility of findings within the community and ensures that they resonate with everyday realities. With access to such data, communities can be better equipped to identify systemic inequities, make visible the ways in which institutions fail to meet their needs, and advance arguments for change that are both empirically supported and experientially valid.

Data can also be used to build collective power to confront systems and demand change, serving as a catalyst for social organizing and mobilization. The term mobilization refers to the process through which a group shifts from being a passive collection of individuals to becoming active participants in public life (Tilly, 2017). Community mobilization is widely recognized as a powerful strategy for achieving sustainable social change by positioning families and local actors as active participants rather than passive recipients of services (Campbell, 2014). Within the field of early childhood education (ECE), this approach highlights that access and quality are not simply matters of policy design or service availability, but also of cultural fit, shared ownership, and responsiveness to community voices. When families and communities mobilize around ECE, they can define what “accessibility” truly means in their context—whether it involves bilingual teachers, extended program hours, or the integration of cultural traditions into classroom practice (Mendez and Crosby, 2024), and so on.

#### Drawing on community organizing frameworks to build power

In our research partnership between the Family Council and the university, we have intentionally drawn on a community organizing framework (Fields et al., 2022) to build and leverage the Family Council’s power within the ECE space. Through ongoing, collaborative data collection efforts—particularly those connected to the development of a tool designed to support families in navigating the ECE system—members of the Family Council are not only generating actionable knowledge but are simultaneously strengthening their organizational capacity and collective influence. This approach reflects core principles of community organizing: developing leadership among community members, fostering shared analysis of systemic barriers, and creating opportunities for families to take strategic action toward change. As families engage in collecting, interpreting, and using data, they cultivate both *people power*—the knowledge, skills, and confidence to advocate effectively—and *organizational power*—the capacity to coordinate, mobilize, and sustain collective efforts. By integrating research and organizing in this way, we are working toward a model in which families themselves drive the strategies and priorities that can shift policies, practices, and systems in early care and education, ensuring that change is community-led and sustainable. Building on this foundation of power and capacity, families and community leaders can use the co-generated findings to take strategic action within their communities and the broader policy landscape. By translating data into accessible and engaging formats—such as community briefs, gallery walks, or storytelling paired with quantitative results—they can highlight structural barriers and inequities in areas like housing, food access, or early care and education. This approach positions families not only as interpreters of knowledge but as active agents who can convene public forums, meet with policymakers, or lead advocacy campaigns that press systems to respond. When communities guide how data are used and shared, they can shift power away from academic or institutional gatekeepers, mobilize collective will, and organize to advance more equitable and responsive policies and practices.

## Conclusion

By working in strategic partnership with parents and families as co-producers and disseminators of knowledge regarding navigating and accessing these ECE systems, we democratize knowledge and strengthen communities’ capacity to mobilize for collective action and hold systems accountable. Importantly, participatory and community-engaged research approaches have demonstrated how families lived experiences can generate actionable knowledge that informs policy advocacy, program design, and systems improvement. For example, prior community-based and participatory research initiatives in early childhood and family policy have shown that when families help design research questions, interpret findings, and share results, the work is more likely to surface barriers that traditional research methods overlook and to produce recommendations that are meaningful and actionable for communities and decision makers alike (Vesely et al., 2017, 2021, 2024). These approaches also strengthen trust between researchers and communities while building families’ capacity to engage in advocacy and systems change.

This kind of power building that goes beyond documenting the pain or “brokenness” of communities and instead supports efforts to hold systems accountable to addressing inequities in access to social safety nets (Kapoor, 2019). At the same time, this kind of work requires scholars to build our own capacity to engage in authentic partnerships with communities—to rethink whose knowledge counts, how research agendas are set, and how findings are shared and disseminated. Such commitments will be especially important during these uncertain sociopolitical times.

Our theory of change therefore no longer relies on damage-centered research. Our earlier theory assumed that those in power, when confronted with evidence of harm, suffering, and the intergenerational costs of injustice, would be motivated to make the investments and structural changes needed to remedy those harms. Our new theory of change recognizes that meaningful change occurs when communities are able to build enough power to demand it. In this context, research plays a vital role—not merely as a tool for documenting inequity, but as a mechanism for strengthening collective capacity and supporting communities in their efforts towards their own liberation.
